# Imprinted gene alterations in the kidneys of growth restricted offspring may be mediated by a long non-coding RNA

**DOI:** 10.1080/15592294.2023.2294516

**Published:** 2023-12-21

**Authors:** Thu N. A. Doan, James M. Cowley, Aaron L. Phillips, Jessica F. Briffa, Shalem Y. Leemaqz, Rachel A. Burton, Tania Romano, Mary E. Wlodek, Tina Bianco-Miotto

**Affiliations:** a School of Agriculture, Food and Wine, & Waite Research Institute, University of Adelaide, Adelaide, South Australia, Australia; b Robinson Research Institute, University of Adelaide, Adelaide, South Australia, Australia; c Department of Anatomy and Physiology, The University of Melbourne, Parkville, Victoria, Australia; d SAHMRI Women and Kids, South Australian Health & Medical Research Institute, Adelaide, South Australia, Australia; e College of Medicine and Public Health, Flinders University, Bedford Park, SA, Australia; f Department of Physiology, Anatomy and Microbiology, La Trobe University, Bundoora, Victoria, Australia; g Department of Obstetrics and Gynaecology, The University of Melbourne, Parkville, Victoria, Australia

**Keywords:** Intrauterine growth restriction, uteroplacental insufficiency, epigenetic mechanisms, long non-coding RNA, DNA methylation

## Abstract

Altered epigenetic mechanisms have been previously reported in growth restricted offspring whose mothers experienced environmental insults during pregnancy in both human and rodent studies. We previously reported changes in the expression of the DNA methyltransferase *Dnmt3a* and the imprinted genes *Cdkn1c* (Cyclin-dependent kinase inhibitor 1C) and *Kcnq1* (Potassium voltage-gated channel subfamily Q member 1) in the kidney tissue of growth restricted rats whose mothers had uteroplacental insufficiency induced on day 18 of gestation, at both embryonic day 20 (E20) and postnatal day 1 (PN1). To determine the mechanisms responsible for changes in the expression of these imprinted genes, we investigated DNA methylation of KvDMR1, an imprinting control region (ICR) that includes the promoter of the antisense long non-coding RNA *Kcnq1ot1* (*Kcnq1* opposite strand/antisense transcript 1). *Kcnq1ot1* expression decreased by 51% in growth restricted offspring compared to sham at PN1. Interestingly, there was a negative correlation between *Kcnq1ot1* and *Kcnq1* in the E20 growth restricted group (Spearman’s *ρ =* 0.014). No correlation was observed between *Kcnq1ot1* and *Cdkn1c* expression in either group at any time point. Additionally, there was a 11.25% decrease in the methylation level at one CpG site within KvDMR1 ICR. This study, together with others in the literature, supports that long non-coding RNAs may mediate changes seen in tissues of growth restricted offspring.

## Introduction

Development is susceptible to environmental insults, such as uteroplacental insufficiency, maternal suboptimal diets, and other environmental exposures to chemicals, infections, drugs, and alcohol [1–7]. Developmental environmental exposure early in life has been shown to be associated with epigenetic changes, including changes in DNA methylation, histone modifications, long non-coding RNA (lncRNA), and micro-RNA (miRNA) expression, in both human and rodent studies, which can have a significant impact on short- and long-term offspring health [1,4–6,8–10]. Additionally, altered epigenetic mechanisms and physiology due to environmental exposure during gametogenesis/gestation have been reported to have multigenerational or transgenerational effects that occur in a sex-specific manner in rodent studies [4–7,9–17]. These animal models have been suggested to be more appropriate for transgenerational studies, as besides the availability of tissues for sampling, inbred strains and strictly controlled experimental environments can help reduce biases found in human studies, such as genetic, ecological, and cultural factors [18].

We have recently shown in our rodent model of uteroplacental insufficiency (UPI) that the expression of *Dnmt3a*, a *de novo* DNA methyltransferase, but not *Dnmt1*, whose primary role is maintaining the DNA methylation landscape, was decreased in the kidney of embryonic day 20 (E20) offspring, which is during the embryonic nephron formation period [12]. Concurrently, expression of imprinted genes that are known to be important in kidney development, *Cdkn1c* and *Kcnq1*, was also altered at both E20 (*Cdkn1c*; sex-specific) and postnatal day 1 (PN1; *Cdkn1c* and *Kcnq1*) [12]. Specifically, at E20, *Cdkn1c* expression was only reduced in growth restricted females. At PN1, regardless of sex, *Cdkn1c* expression was lower and *Kcnq1* expression was higher in growth restricted offspring, in association with reduced absolute and percentage left kidney weight [12]. Interestingly, *Kcnq1* and *Cdkn1c* are both known to be regulated by KvDMR1, an imprinting control region (ICR), which includes the promoter of the imprinted antisense lncRNA *Kcnq1ot1* [19,20]. These results raised a question of whether epigenetic mechanisms, such as DNA methylation or lncRNAs, can explain the multigenerational and sex-specific alterations in both gene expression and growth phenotypes in the kidneys of growth restricted offspring.

In the current study, we investigated the relationship between *Kcnq1* and *Cdkn1c* with *Kcnq1ot1* and KvDMR1 by examining the expression of *Kcnq1ot1* and the DNA methylation status of two CpG islands within the KvDMR1 ICR in the kidneys of F1 growth restricted offspring. The study will contribute to the understanding of the potential mechanisms controlling the gene expression of imprinted genes in the kidney that might be susceptible to adverse *in utero* environments.

## Materials and methods

### Kidney tissue collection

The intrauterine growth restricted (IUGR) Wistar Kyoto rat model was generated as previously described (The University of Melbourne AEC 04138, 1011865, and 1112130; La Trobe University AEC 12–42) [12,21,22]. In short, pregnant female rats (F0) underwent bilateral uterine vessel (artery and vein) ligation at day 18 of pregnancy (late gestation; term = 22 days) to induce UPI. The control group underwent sham surgery (no vessel ligation). Left kidney samples were collected at embryonic day 20 (E20) and post-natal day 1 (PN1) from the first-generation rat offspring, with one male and one female examined per litter [12]. Samples were snap frozen in liquid nitrogen and stored at −80°C.

### RNA and DNA extraction

RNA was extracted from samples as described previously [12]. For DNA extraction, 30 mg of left kidney tissue was quickly cut on a plastic weight boat on ice. Only PN1 tissues were available for DNA extraction as the whole E20 kidney was used in RNA extraction [12]. Tissue homogenization was carried out in 500 µL of TES (10 mM Tris (pH 8.0), 1 mM EDTA, 0.1 M NaCl; Invitrogen) with the following PowerLyser settings: time ‘T’ = 15 s, cycles ‘C’ = 1, dwell/pause time ‘D’ = 0 s, and speed ‘S’ = 3,500 rpm. DNA was then extracted using the salting out method [23] with modifications. Thirty microlitres of 20 µg/µL Proteinase K (Invitrogen) was added to each tube of homogenized tissue (mixed by inversion), followed by 60 µL of 20% SDS (Invitrogen) (mixed by inversion). The samples were then incubated at 37°C for 24 h. After incubation, 300 µL of 3 M NaCl was added to each tube and mixed vigorously by shaking for at least 10 s. Tubes were placed on ice for 10 min, followed by centrifugation at 13,000 rpm for 15 min, and a maximum of 450 µL of the supernatant was collected. Two microlitres of glycogen (Invitrogen) was added to each tube, followed by 900 µL of 100% molecular biology grade ethanol (Sigma-Aldrich) (mixed by inversion). The DNA was pelleted by centrifugation at 13,000 rpm for 2 min. The DNA pellet was washed with 900 µL 70% ethanol (mixed by inversion) and centrifuged at 13,000 rpm for 1 min. The supernatant was then removed, and the DNA pellet was centrifuged at 13,000 rpm for 1 min. The DNA pellet was dried at room temperature before resuspension in TE buffer (Invitrogen) (50 µL, pH 8.0). Samples were stored at 4°C, and the DNA concentration was quantitated using a NanoDrop spectrophotometer (Thermo Fisher Scientific). DNA integrity was checked using 1% agarose gel electrophoresis.

### Genomic DNA (gDNA) contamination check and reverse transcription

RNA samples (20 ng, in duplicate) were checked for contamination of gDNA as previously described [12] using the SsoAdvanced™ Universal SYBR® Green Supermix (Bio-Rad) and primers that targeted an *Actb* intronic region. Contaminated RNA samples (Cq < 35) were DNase-treated using the TURBO DNA-freeTM kit (Thermo Fisher Scientific) and checked again using the same qPCR method.

### qPCR gene expression analysis

*Tbp* and *Ywhaz* were determined to be the two most stable reference genes in our previous study [12]. As the lncRNA *Kcnq1ot1* sequence is not available on the rat assembly (UCSC Genome Browser Nov. 2020 (mRatBN7/rn7)), *Kcnq1ot1* sequence from the mouse genome (UCSC Genome Browser Jun. 2020 (GRCm39/mm39)) was submitted to a UCSC BLAT search against the rat genome. Primers for *Kcnq1ot1, Slc22a18*, and *Cars* were then designed using NCBI Primer-BLAST (Table S1). Primer optimization, master mix preparation and qPCRs were performed as previously described [12], with cycling conditions shown in Table S1.

### DNA methylation analysis

A total of 34 rat PN1 DNA samples (1000 ng each) were sent to the Australian Genome Research Facility (AGRF) for region-specific quantitative DNA methylation analysis. Primers targeting two CpG islands (chr1:198,492,806–198,493,065 (CpG: 23) and chr1:198,493,269–198,493,580 (CpG: 20) (mRatBN7/rn7)) on the KvDMR1 imprinting control region were designed by AGRF (Table S2). DNA samples were bisulphite modified, followed by analyses using EpiTYPER Agena MassArray and Mass Cleave Chemistry test methods [24].

### Data analysis

Data were analysed using a linear mixed-effect model, with adjustments for litter size and relatedness between litter siblings as previously reported [12], using R version 4.1.1 [25,26]. Power of the linear mixed-effect model was determined to be 0.998 and 0.993 for the analysis of gene expression and DNA methylation, respectively, calculated using the ‘pwr.f2.test’ function (‘pwr’ package) in the R environment, with n (sample size) = 38 for our expression studies and *n* = 33 for the DNA methylation analyses, respectively. Correlation between gene expression levels were determined using Spearman’s non-parametric correlation coefficient (no assumptions regarding data distribution), calculated using PAST 4.03 software [27]. Sham and IUGR data were combined to investigate whether there is a relationship between expression of different pairs of genes, regardless of treatment. The relationships within each group were then examined to explore whether a certain correlation is present in one group and is absent/altered in the other group, potentially indicating disruption due to growth restriction.

## Results

### Expression of imprinted and non-imprinted genes in the kidney

**Figure 1.**
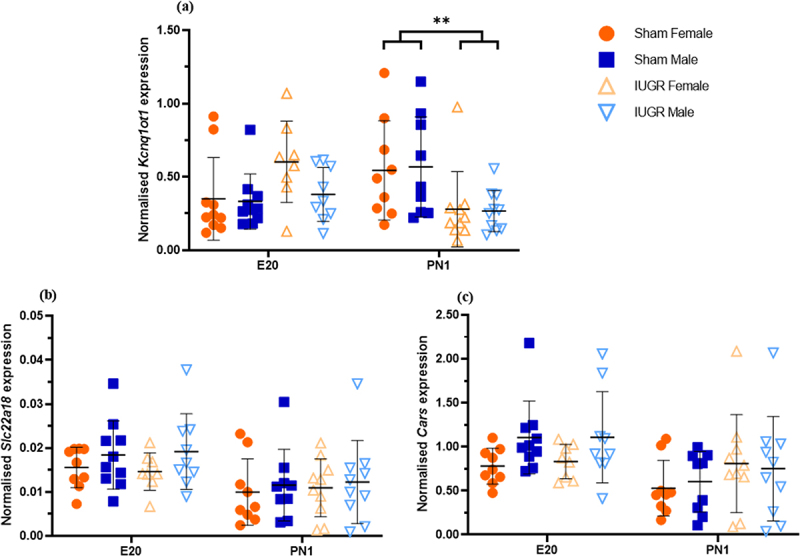
Normalised expression of the imprinted genes *Kcnq1ot1* (a), *Slc22a18* (b), and the non-imprinted gene *Cars* (c) in kidney tissues of sham and IUGR rat offspring at embryonic day 20 (E20) and postnatal day 1 (PN1). Significance was determined by linear mixed effect models, followed by a Tukey’s *post hoc* test (**p < 0.01). Data is expressed as mean ± SD; *n* = 8–10/group.

The expression of *Kcnq1ot1* was not different between the sham and IUGR offspring at E20 (Figure 1a). However, at PN1, there was a significantly lower expression of *Kcnq1ot1* in IUGR offspring than in sham offspring (reduced by approximately 50%, *p <* 0.01). The expression of another imprinted gene in the same KvDMR1 ubiquitously imprinted cluster (*Slc22a18*) and a non-imprinted gene (*Cars*) was also examined to determine whether the changes observed in *Kcnq1ot1*, *Kcnq1* and *Cdkn1c* extended to other genes in this imprinting cluster. There was no significant difference in the expression of either *Slc22a18* (Figure 1b) or *Cars* (Figure 1c) between the sham and IUGR offspring at any time point.

### Correlation between gene expression levels in rat kidney

**Figure 2.**
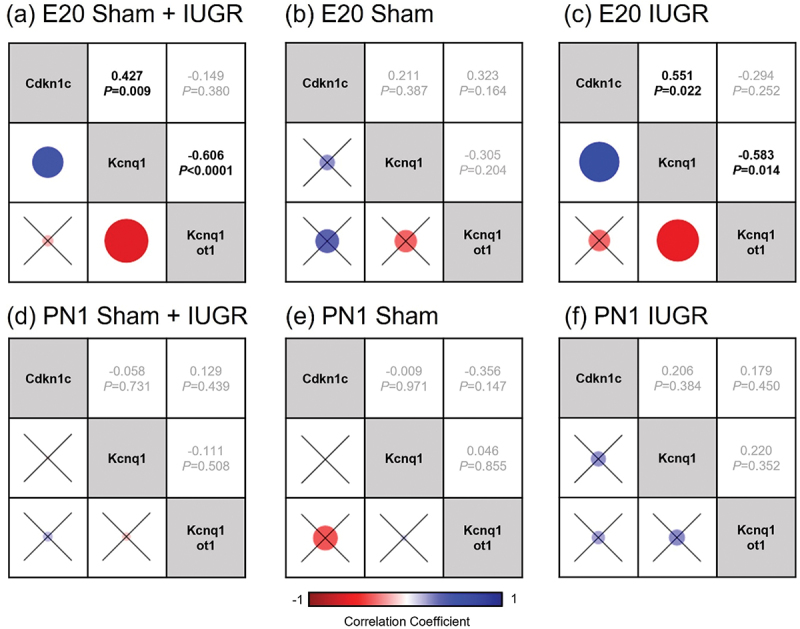
Spearman’s non-parametric correlation matrices between three imprinted genes known to be important in kidney development and regulated by the KvDMR1 imprinting control region (*Kcnq1ot1*, *Cdkn1c* and *Kcnq1*) in kidney tissues of sham and IUGR rat offspring at embryonic day 20 (E20) and postnatal day 1 (PN1). Sham and IUGR data were combined in (a) for E20 and (d) for PN1. Spearman correlation coefficients (top number) and *p*-values (bottom number) are displayed on the right triangles. A cross through the box indicates a non-significant *p*-value. The size of the circle indicates how strong the correlation is (corresponded to the Spearman correlation coefficients).

Pairwise non-parametric correlation analyses were carried out to investigate the potential correlations between the expression levels of genes in sham and IUGR offspring at E20 and PN1, including between pairs of imprinted genes known to be important in kidney development and regulated by the KvDMR1 ICR (*Cdkn1c*, *Kcnq1,* and *Kcnq1ot1*; Figure 2 and Table S3), as well as between imprinted genes and other genes (*Dnmt1a*, *Dnmt3a*, *Peg3*, *Snrpn*, *Slc22a18*, and *Cars*; Fig. S1 and Table S3). The expression of *Cdkn1c*, *Kcnq1*, *Dnmt1a*, *Dnmt3a*, *Peg3,* and *Snrpn* has been previously reported [12].

When sham and IUGR data were combined, there was a significant negative correlation between the expression of *Dnmt3a* and lncRNA *Kcnq1ot1* at E20 (Spearman’s ρ = −0.455, *p =* 0.006, Fig. S1a and Table S3), as well as significant positive correlations between *Dnmt3a* and *Kcnq1* and *Dnmt3a* and *Cdkn1c* (Spearman’s ρ = 0.896, *p <* 0.0001 and Spearman’s ρ = 0.349, *p =* 0.040, respectively; Fig. S1a and Table S3). Additionally, at E20, there was a negative correlation between *Kcnq1ot1* and *Kcnq1* and a positive correlation between *Kcnq1* and *Cdkn1c* (Spearman’s ρ = −0.606, *p <* 0.0001 and Spearman’s ρ = 0.427, *p =* 0.009, respectively; Figure 2a and Table S3). The relationships between these pairs of genes (except *Dnmt3a-Kcnq1*) were no longer present at PN1 (Fig. S1b and Table S3).

Interestingly, when sham and IUGR were investigated individually at each time point, the negative correlation between *Kcnq1ot1* and *Kcnq1* was significant only in the E20 IUGR group (Spearman’s ρ = −0.583, *p =* 0.014, Figure 2c–f, S1c-f and Table S3). Additionally, there was a significant positive correlation between *Kcnq1* and *Cdkn1c* in the E20 IUGR group (Spearman’s ρ = 0.551, *p =* 0.022), but not in the E20 sham group (Figure 2b,c, S1c, S1e and Table S3). No correlation was observed between *Kcnq1ot1* and *Cdkn1c* expression in any of the groups at any time point. On the other hand, there was an inverse relationship between *Dnmt3a* and *Kcnqot1* in the IUGR group, whereby at E20, there was a negative association (Spearman’s ρ = −0.421) and at PN1, there was a positive association (Spearman’s ρ = 0.370) (Fig. S1e, S1f and Table S3). However, these differences were not statistically significant.

### DNA methylation status of the KvDMR1 imprinting control region

**Figure 3.**
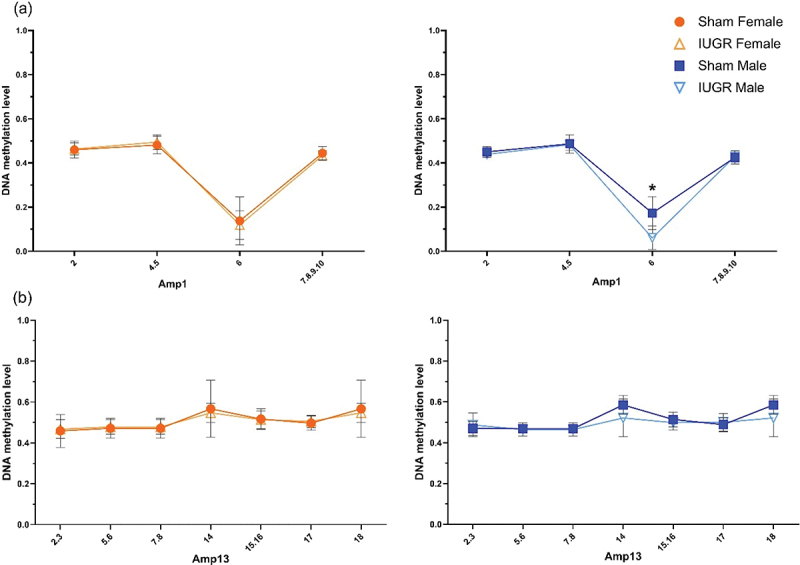
DNA methylation status of the KvDMR1 imprinting control region containing (a) CpG 23 (amplicon 1 (amp1), chr1:198,492,806–198,493,065, UCSC genome Browser Nov. 2020 (mRatbn7/rn7)) and (b) CpG 20 (amplicon 13 (amp13), chr1:198,493,269–198,493,580 (mRatbn7/rn7)) in sham and IUGR rat offspring at postnatal day 1 (PN1), determined using EpiTYPER Agena MassArray and mass cleave chemistry analyses. For CpG fragments that had the same mass peaks as other fragments containing same number of CpGs (Amp13, CpG_5.6 versus CpG_7.8 and CpG_14 versus CpG_18), methylation % was calculated between CpGs. Significance was determined by linear mixed effect models, followed by a Tukey’s *post hoc* test (*p < 0.05). Data is expressed as mean ± SD; *n* = 8–9/group.

Base-specific cleavage of bisulphite-modified DNA yielded usable signals for four out of 16 (amplicon 1, Figure 3a) and seven out of 20 (amplicon 13, Figure 3b) CpG positions within CpG 23 and CpG 20 islands, respectively, in KvDMR1 ICR. There was hypomethylation (*p* < 0.05) at CpG site 6 of the CpG 23 island in IUGR males only (↓11.25%, methylation level in IUGR males 6% *vs*. sham males 17.25%, amplicon 1, Figure 3a). Interestingly, unlike other CpG sites within this region where the DNA methylation level was ~50% (as expected for imprinted genes), there was a lower than 20% methylation level at CpG site 6, even in the sham animals. There was no statistically significant difference in the methylation status between sham and IUGR offspring at any site of the CpG 20 island (amplicon 13, Figure 3b).

**Figure 4.**
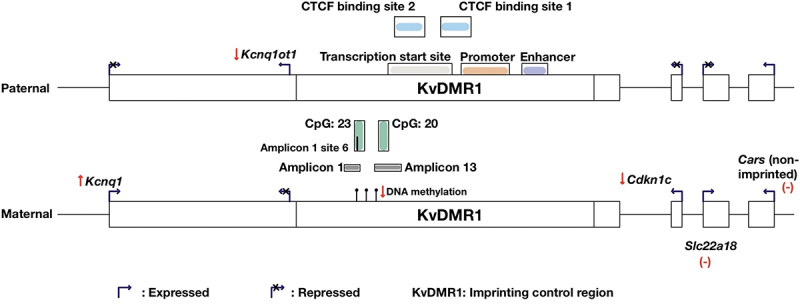
Approximate positions of the two amplicons (amplicon 1 and 13, targeting CpG island 23 (chr1:198,492,806–198,493,065) and 20 (chr1:198,493,269–198,493) (mRatbn7/rn7), respectively) in the rat KvDMR1 imprinting control region (modified from Doan *et al* [12].), examined using region-specific quantitative DNA methylation analysis. DNA methylation of KvDMR1 and/or expression of the lncRNA *Kcnq1ot1* is known to play a role in controlling the monoallelic expression of imprinted genes in the KvDMR1 imprinting cluster. Primers were designed by the Australian genome Research Facility (AGRF). There was a hypomethylation (↓11.25%, *p* < 0.05) at CpG site 6 of CpG 23 island in PN1 growth restricted male kidneys. *Kcnq1ot1* sequence is not available on the mRatbn7/rn7 rat genome. Hence, sequence from the uncharacterized lncRNA named LOC120099961 found on the rat mRatbn7.2 genome (NCBI reference sequence: NC_051336.1, similar position) was used. Mouse sequences, including KvDMR1 region [28], *Kcnq1ot1* transcriptional repressor CTCF binding sites [29], enhancer, promoter [30], and transcription start site [30,31] were used in a BLAT search against the rat genome. ↓: expression decreased; ↑: expression increased; (-): no change in gene expression. Note that the annotations of gene expressions in this figure is based on the circumstance that in a healthy animal, the imprinted genes *Kcnq1* and *Cdkn1c* are expressed on the maternal allele, while *Kcnq1ot1* is preferentially expressed on the paternal allele.

KvDMR1 ICR was further analysed to identify the location of these CpG sites. As mentioned previously, the *Kcnq1ot1* sequence is not available in the mRatBN7/rn7 rat genome. However, there was an uncharacterized lncRNA named LOC120099961 found in the rat mRatBN7.2 genome (NCBI Reference Sequence: NC_051336.1), which is located in a similar position as *Kcnq1ot1* in other species genomes. Therefore, this rat sequence, together with other mouse sequences including the KvDMR1 region [28], *Kcnq1ot1* transcriptional repressor CTCF binding sites [29], enhancer, promoter [30], and TSS [30,31] were used in a BLAT search against the rat genome. The results for the (approximate) positions are shown in Figure 4. While amplicon 13 (CpG 20) was located within both *Kcnq1ot1* TSS and CTCF binding site 2, amplicon 1 (CpG 23) was not located within any of the sequences mentioned above (Figure 4). Using TFBIND software (weight matrix in transcription factor database TRANSFAC R.3.4, similarity ≥80%) [32] and TRANSFAC FACTOR TABLE (Release 2017.2), CpG site 6 (amplicon 1) was determined to correspond to different transcription factor binding sites (TFBSs) (Table 1). Among these, there were four TF that have been previously reported to play a role in kidney development and disease, as well as to be regulated by DNA methylation, including Chicken Ovalbumin Upstream Promoter Transcription Factor 2 (COUP-TF2) [33–36], GATA-binding Factor 2 (GATA-2) [37,38], Serum Response Factor (SRF) [39–41], and Activating enhancer binding Protein 2 alpha (AP-2α) [42,43]. When data from all examined CpG sites within each CpG island were combined, no significant difference in DNA methylation levels was found between the sham and IUGR kidney samples (Fig. S2).

**Table 1.** Transcription factor binding sites (TFBSs) correspond to CpG:23 island (amplicon 1, chr1:198,492,806–198,493,065), where there was a hypomethylation at CpG site 6 (coloured in red) in IUGR male kidneys. TFBSs were determined using TFBIND software (weight matrix in transcription factor database TRANSFAC R.3.4) [32] and TRANSFAC FACTOR TABLE (release 2017.2). Left to right: TF name, matrix ID (from TRANSFAC R.3.4), label in TFBIND, similarity compared to input sequence, strand that the transcription factor binds, and sequence of the TFBS.

Factor	ID	Label	Similarity	Forward (+) or reverse (-)	Sequence
COUP-TF2	M00155	ARP1_01	0.806	(+)	CGCGGCCATGAAACG^6^C
GATA-2	M00076	GATA2_01	0.806	(-)	CG^6^CCAACCGG
SRF	M00215	SRF_C	0.803	(+)	GCCATGAAACG^6^CCAA
AP-2α	M00189	AP2_Q6	0.801	(+)	CG^6^CCAACCGGGC

## Discussion

The imprinted gene *Kcnq1ot1* has been previously shown to be altered in growth restricted offspring due to environmental exposure during early life [44,45]. Specifically, reduced expression of this lncRNA has been reported in placentae of E16.5 growth restricted male mice whose mothers were exposed to 50 ppm of the heavy metal cadmium throughout pre-conception, mating, and pregnancy [44], as well as in E18.5 growth restricted mice who were conceived through *in vitro* fertilization (IVF) [45]. In our current study, as expected, there was a significant decrease in *Kcnq1ot1* expression in kidneys of F1 growth restricted rat offspring at PN1. From studies in mice, the function of *Kcnq1ot1* is suggested to partially control the allele-specific expression of other imprinted genes in the same KvDMR1 imprinting cluster, including those investigated in this current study, in a tissue-specific manner; however, the exact mechanism is still unclear [29,46,47]. For instance, deletion of the whole KvDMR1 ICR (2.8 kb [46] or 3.6 kb [47], which abolished *Kcnq1ot1* expression), deletion of *Kcnq1ot1* promoter and TSS region (224 bp) [47], producing a shorter transcript by inserting a transcription stop element at 1.5 kb downstream of the lncRNA TSS [47], or truncation of *Kcnq1ot1* (2.6 kb downstream of its promoter) [29], on the paternal allele, was reported to be associated with activation of the normally paternally silenced genes in mouse embryonic tissues (E11.5–16.5). Biallelic gene expression was reported for *Slc22a18* (placenta [29,47], liver, gut, kidney, lung, heart, brain, and fibroblast [29]), *Kcnq1* (placenta [29,47], liver [29,46], gut, kidney, lung, heart, brain, and fibroblast [29]), and *Cdkn1c* (whole embryo, placenta [29,47], liver [46], heart, brain, and gut [29]). However, monoallelic expression of *Cdkn1c* has been reported in the liver, kidney, lung, and fibroblasts of mice at E15.5, despite the *Kcnq1ot1* truncation, which remains to be explained [29].

In line with the above findings, studies in mouse IUGR models also reported alterations to the imprinted genes that are known to be regulated by KvDMR1, in association with decreased *Kcnq1ot1* expression [44,45]. Growth restricted mice conceived through IVF have decreased placental *Cdkn1c* expression compared to *in vivo* controls at E18.5, despite a similar expression at E14.5 [45]. In contrast, *Cdkn1c* overall expression was increased in the placentae of E18.5 growth restricted mice whose mothers were exposed to Cadmium [44]. Meanwhile, there was no alteration in placental *Kcnq1* expression in these mice [44]. Additionally, allele-specific expression analysis indicated no difference in *Cdkn1c* expression between growth restricted and sham animals [44]. In our study of growth restricted rat kidneys, *Cdkn1c* expression was reduced only in IUGR females at E20, while PN1 IUGR offspring had decreased *Cdkn1c* and increased *Kcnq1* expression compared to sham [12]. Together with the above-mentioned findings, the fact that our results report decreased *Kcnq1ot1* only in PN1, but not E20, IUGR rats as well as no correlation between *Kcnq1ot1* and *Cdkn1c* expression in any of the groups, at any timepoint, suggests that changes in lncRNA *Kcnq1ot1* expression alone is not sufficient to explain changes in *Cdkn1c* in IUGR rat kidneys. Allele-specific expression analysis of these imprinted genes would provide a better understanding of their potential relationships.

As *Dnmt3a* was reported in our previous study to be decreased in IUGR kidneys at E20 [12], we hypothesized that there were alterations in the DNA methylation profile, including that of the KvDMR1 ICR, which is involved in dysregulation of the expression of imprinted genes that are known to be important in foetal kidney development. In babies diagnosed with Russell-Silver syndrome, characterized by intrauterine and postnatal growth restriction, alterations in KvDMR1 DNA methylation, either hypermethylation [48–50] or hypomethylation [51], have been reported in their blood samples. In human IUGR studies, KvDMR1 DNA methylation status was mostly studied using placental tissues, with no significant difference observed between growth restricted tissues and healthy controls [52–55]. In the current study of rat kidneys, hypomethylation was found at a CpG site of CpG 23 island (chr1:198,492,806–198,493,065) within KvDMR1 in PN1 IUGR males. This CpG site was not located within any of the *Kcnq1ot1* regulatory regions that we were able to assess. However, this position is a potential target for several TFs known to be important in kidney development and disease, including but not limited to COUP-TF2, GATA-2, SRF, and AP-2α. Future studies should investigate the potential interaction of these TFs with KvDMR1 and the biological function of such event. Furthermore, as these TFs have been previously shown to be impacted by DNA methylation [33,34,38,39,42], alteration to the *Dnmt3a* expression in our study could also have an effect on their expression and/or function. Another important point to mention here is that DNA methylation level of this specific site was also lower than 50% in sham animals, which is not typical for imprinted genes where the silenced allele is often methylated. Meanwhile, investigation of the CpG 41 island in the placentae of E18.5 growth restricted female mice (conserved sequence of KvDMR1 CpG 23 island in rats) showed no change in DNA methylation of any other CpG sites within this region (chr1:198,493,086–198,493,233) [44]. In addition, our results show that the mean DNA methylation levels within this CpG 23 island as well as within the CpG 20 island (chr1:198,493,269–198,493,580) of the KvDMR1 ICR were also not different between sham and IUGR offspring. Nonetheless, apart from the differences in tissues examined, it should be noted that different regions within and near the KvDMR1 ICR were investigated in the above studies, which could be a potential limitation of the present study. Additionally, the kidney is a complex organ that comprises more than 20 differentiated cell types [56]. Recent single-cell RNA sequencing databases in both adult mice [57,58] and rats [59] suggest that the three imprinted genes (*Cdkn1c*, *Kcnq1*, and *Kcnq1ot1*) investigated in our study have different expression levels in different renal cell types. Specifically, *Cdkn1c* is highly expressed in stromal cells and podocytes (visceral epithelium), while *Kcnq1* is highly expressed in collecting duct intercalated cells and connecting tubule principal-like cells. *Kcnq1ot1* (mouse data) is also highly expressed in podocytes. Since we only assessed DNA methylation of one region using region-specific quantitative DNA methylation analysis method, this did not allow for assessing or adjusting for different cell types.

Besides KvDMR1, DNA methylation of the *Cdkn1c* promoter region is also an important mechanism that needs to be explored, as it is known to be important in maintaining allele-specific gene expression during embryonic development in healthy mice [60]. However, in the mouse *Kcnq1ot1* truncation model, where *Cdkn1c* allele-specific expression was shown to be either altered or unchanged in different embryonic tissues, there was no difference in *Cdkn1c* promoter DNA methylation levels in all tissues at E15.5, suggesting a different mechanism for maintaining *Cdkn1c* monoallelic expression [29]. In contrast, in the placentae of E18.5 Cadmium-exposed growth restricted mice, where expression of *Kcnq1ot1* decreased and expression of *Cdkn1c* increased, there was a reduction in DNA methylation in one out of 23 investigated CpG sites in the *Cdkn1c* promoter region [44]. However, the mean methylation level of the whole CpG island did not change compared with that of the sham offspring [44]. Future studies should investigate epigenetic alterations in the *Cdkn1c* promoter region.

In summary, at PN1, there was a 50% decrease in the expression of an antisense lncRNA (*Kcnq1ot1*) in IUGR rats compared to that in sham animals. This is the first study to report changes in *Kcnq1ot1* in UPI-induced growth restricted rat kidneys. *H19* is another lncRNA and imprinted gene that plays an important role in development. *H19* has also been shown to be altered in rodent and human IUGR studies, with significant changes in its expression and DNA methylation in many tissues (e.g., sperm, liver, blood, and placenta [1,61–63]). In this study, there was a negative correlation between *Kcnq1ot1* and the gene that it is located within (*Kcnq1*), only in E20 IUGR kidneys. As *Kcnq1* was also altered at PN1 [12], these results suggest that an abnormal event occurred early during foetal nephron formation, which later affected the expression of imprinted genes within the KvDMR1 ICR. In contrast, changes in *Kcnq1ot1* were not sufficient to explain the decrease in the expression of another imprinted gene within the same KvDMR1 imprinting cluster, *Cdkn1c*, at both E20 (IUGR females) and PN1 (IUGR males and females) [12], as no correlation was found between the two genes in any group at any time point. As there was a decrease in *Dnmt3a* expression in E20 IUGR kidneys [12] and significant correlations between *Dnmt3a* and *Kcnq1/Kcnq1ot1/Cdkn1c* at E20, the DNA methylation profile of KvDMR1 was investigated. Hypomethylation was found at a CpG site only in PN1 IUGR males. However, the importance of the alteration of this specific CpG site and its effect on the IUGR kidney is yet to be determined. Future studies should investigate the allele-specific expression of these genes, the reason for DNA methylation changes at one CpG site in KvDMR1, and other epigenetic mechanisms.

## Disclaimers

The views expressed in this manuscript are those of the authors.

## Supplementary Material

Supplemental Material

## Data Availability

The data that support the findings of this study are available from the corresponding author (tina.bianco@adelaide.edu.au) upon request.
