# Supplementary material for: Imprinted gene alterations in the kidneys of growth restricted offspring may be mediated by a long non-coding RNA
Source: Epigenetics. 2023 Dec 21;19(1):2294516. doi: 10.1080/15592294.2023.2294516 (PMC10761017; doi:10.1080/15592294.2023.2294516)
Supplement: Supplemental Material [file KEPI_A_2294516_SM0673.docx]

**Figure S1.**

**
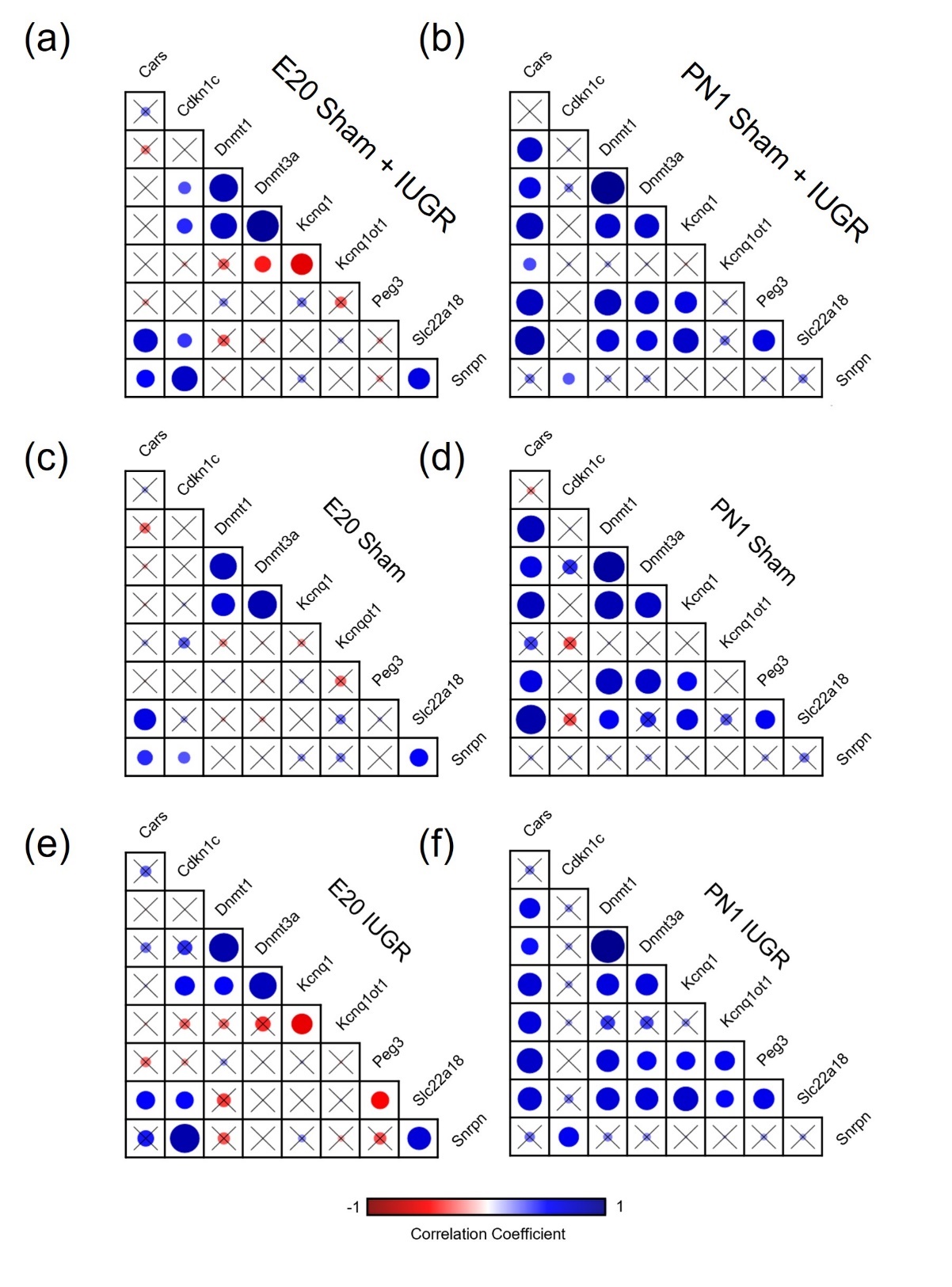
**

**Figure S2.**


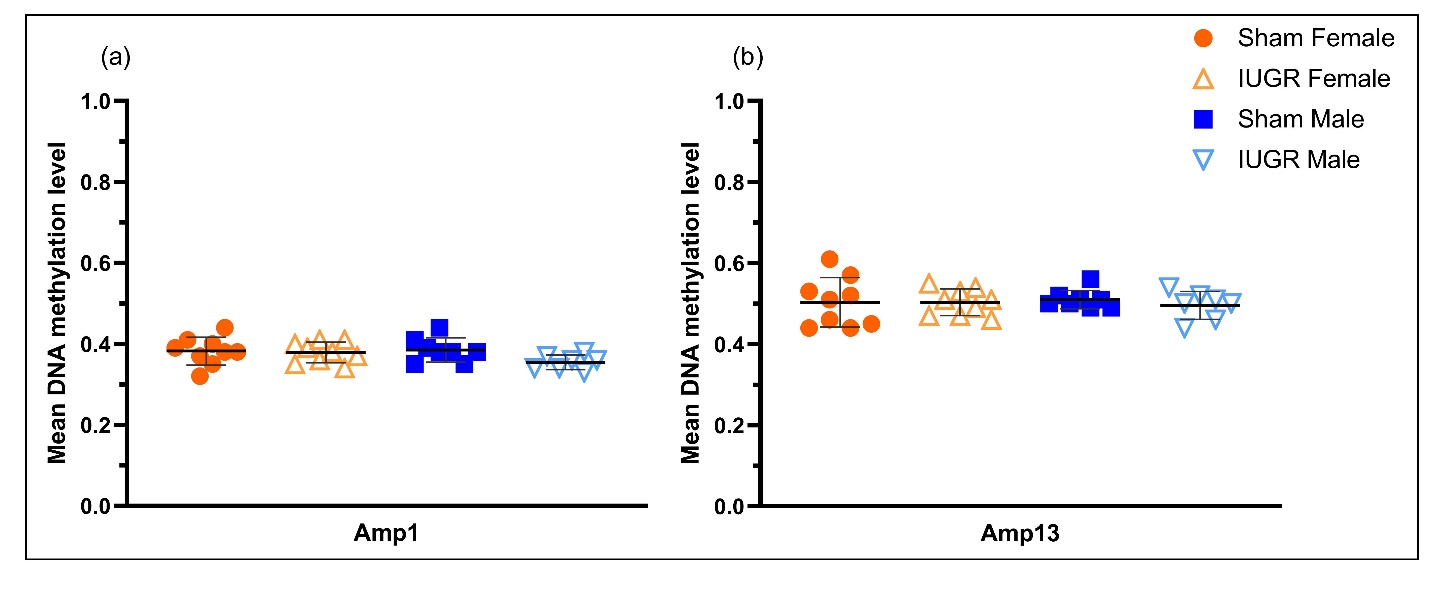


**Table S1.** Primer sequences for the rat reference (*Tbp* and *Ywhaz*), imprinted (*Kcnq1ot1* (long non-coding RNA), *Slc22a18*), and non-imprinted (*Cars*) genes. Primers were optimised at the following qPCR cycling conditions: 98^o^C for 3 minutes, (98^o^C for 10 seconds, 60 or 63^o^C for 30 seconds (*)) – repeated for 40 cycles, followed by melt curve analysis: 65^o^C to 90^o^C with 0.5^o^C increment per 5 seconds.

| **Gene** | **Primers** | **Sequence (*5’* to *3’*)** | **Primer length** | **PCR product length (bp)** | **Annealing temperature (*)** |
| --- | --- | --- | --- | --- | --- |
| *Tbp* | *RTTbpF* | CTAACCACAGCACCATTG | 18 | 152 | **63^o^C** |
|  | *RTTbpR* | TTACAGCCAAGATTCACG | 18 |  |  |
| *Ywhaz* | *RTYwhazF* | ACCCACTCCGGACACAGAAT | 20 | 111 | **63^o^C** |
|  | *RTYwhazR* | GACTTCATGCAGGCTGCCA | 19 |  |  |
| *Kcnq1ot1* | *RTKcnq1ot1F* | AAAATGAAAAGGGTGAGACATGG | 23 | 150 | **63^o^C** |
|  | *RTKcnq1ot1R* | TCACAAATTTGGTTTTTCTACCCA | 24 |  |  |
| *Slc22a18* | *RTSlc22a18F* | CTCTTCGCCTCGCGTCTAC | 19 | 152 | **60^o^C** |
|  | *RTSlc22a18R* | AGCAGGGAGCCGAAGATAAC | 20 |  |  |
| *Cars* | *RTCarsF* | ATCGGGAGCAGAAACCTTCG | 20 | 152 | **60^o^C** |
|  | *RTCarsR* | TGGTTCTGTGGCAAGCTTCA | 20 |  |  |

**Table S2.** Primer sequences for DNA methylation analysis of two CpG islands (chr1:198,492,806 - 198,493,065 (CpG: 23, Amp_1) and chr1:198,493,269 - 198,493,580 (CpG: 20, Amp_13) (mRatBN7/rn7)) on the rat KvDMR1 imprinting control region. Primers were designed by the Australian Genome Research Facility (AGRF) using EpiDesigner (Agena Bioscience). Blue texts denote CpG site positions that were investigated.

| **Amplicon name** | **Primers** | | **Direction** | **Target sequence** | **Target length** | **Target CpG** | **CpG**  **analysed in T** | **CpG**  **analysed in C** | **Primer C** |
| --- | --- | --- | --- | --- | --- | --- | --- | --- | --- |
| Amp_1 | Left | GTTTAGGGGTTTAATGGATTTTAAG | F | GCTTAGGGGCTCAATGGACCTCAAGACCACCT**CG**^1^GCTTCTGTGAGCCTGGGCTG**CG**^2^AAGATGGAGCCCTGCCTGGGGAGATGTGGCCCAAGGATGAGAAC**CG^3^AGCCG^4^CG^5^GCCATGAAACG^6^**CCAAC**CG**^7^GGC**CG**^8^**CG**^9^GC**CG**^10^TAAAT**CG**^11^AATA**CG**^12^GAGCCCCAAC**CG**^13^CCAAA**CG**^14^AATCC**CG**^15^AGCCACTGTTGCAAAA**CG**^16^AAGATGGAGCCCCAGCCATGGAGGTAAGCAATGGATTCATCTCTGCTTCTGGCCATGTGTGCTTG | 260 | 16 | 11 | 0 | 6 |
|  | Right | CAAACACACATAACCAAAAACAAAA |  |  |  |  |  |  | 7 |
| Amp_13 | Left | GGATTTTGGTTGGTTAAAGAATGTT | F | GGACCCTGGCTGGCTAAAGAATGCTGAGAAGCAAAG**CG**^1^GAG**CG**^2^**CG**^3^CCAAGGCAGC**CG**^4^AC**CG**^5^**CG**^6^CTGGAGAC**CG**^7^**CG**^8^TTGGAGTGATC**CG**^9^TACTGAAATGATCCACACTTAAGTGACC**CG**^10^ATTGCTGAGGTAGATCAGACTGTAG**CG**^11^AGGACCACCATGC**CG**^12^AAACAAGATAAAGACCTCAC**CG**^13^AGGAGGTCTATGCTCAGGAGAAACTGAGGC**CG**^14^AT**CG**^15^**CG**^16^TTGAGCAAAGCACACTGATGATGGCTGGT**CG**^17^GGACTGAGG**CG**^18^CAC**CG**^19^CACTCAAGTGATC**CG**^20^AGCAGAGGCAGATCCAAAAGAATTGTGAAC | 312 | 20 | 18 | 0 | 6 |
|  | Right | ATTCACAATTCTTTTAAATCTACCTCT |  |  |  |  |  |  | 4 |

**Table S3.** Values of correlations matrices displayed in **Figure 2** and **Figure S1**. The lower triangle displays the Spearman correlation coefficients and the upper triangle displays the *p*-value. In the lower triangle, blue text denotes a positive correlation (> 0) while red text denotes a negative correlation (< 0). Significant correlations (*p <* 0.05) are bolded and, in the upper triangle, not greyed out.

| E20 Sham + IUGR | | | | | | | | |
| --- | --- | --- | --- | --- | --- | --- | --- | --- |
| *Cars* | 0.134 | 0.154 | 0.816 | 0.827 | 0.745 | 0.295 | **0.000** | **0.002** |
| 0.255 | *Cdkn1c* | 0.850 | **0.040** | **0.009** | 0.380 | 0.806 | **0.017** | **0.000** |
| -0.250 | 0.033 | *Dnmt1* | **0.000** | **0.000** | 0.071 | 0.177 | 0.061 | 0.497 |
| 0.041 | **0.349** | **0.795** | *Dnmt3a* | **0.000** | **0.006** | 0.705 | 0.375 | 0.554 |
| -0.038 | **0.427** | **0.736** | **0.896** | *Kcnq1* | **0.000** | 0.119 | 0.854 | 0.173 |
| 0.056 | -0.149 | -0.308 | **-0.455** | **-0.606** | *Kcnq1ot1* | 0.051 | 0.326 | 0.713 |
| -0.179 | -0.042 | 0.233 | 0.066 | 0.265 | -0.324 | *Peg3* | 0.313 | 0.263 |
| **0.673** | **0.395** | -0.325 | -0.157 | -0.032 | 0.168 | -0.173 | *Slc22a18* | **0.000** |
| **0.501** | **0.719** | -0.119 | 0.105 | 0.232 | -0.064 | -0.192 | 0.607 | *Snrpn* |

| E20 Sham | | | | | | | | |
| --- | --- | --- | --- | --- | --- | --- | --- | --- |
| *Cars* | 0.318 | 0.130 | 0.295 | 0.526 | 0.254 | 0.808 | **0.000** | **0.016** |
| 0.242 | *Cdkn1c* | 0.737 | 0.814 | 0.387 | 0.164 | 0.821 | 0.218 | **0.046** |
| -0.370 | -0.082 | *Dnmt1* | **0.000** | **0.000** | 0.201 | 0.538 | 0.381 | 0.960 |
| -0.261 | 0.058 | **0.920** | *Dnmt3a* | **0.000** | 0.567 | 0.647 | 0.210 | 0.587 |
| -0.160 | 0.211 | **0.865** | **0.922** | *Kcnq1* | 0.204 | 0.307 | 0.900 | 0.359 |
| 0.275 | 0.323 | -0.307 | -0.140 | -0.305 | *Kcnq1ot1* | 0.083 | 0.096 | 0.156 |
| -0.060 | 0.054 | 0.151 | -0.112 | 0.247 | -0.397 | *Peg3* | 0.424 | 0.753 |
| **0.774** | 0.296 | -0.220 | -0.311 | -0.032 | 0.393 | 0.195 | *Slc22a18* | **0.004** |
| **0.560** | **0.463** | 0.012 | 0.137 | 0.223 | 0.339 | -0.077 | **0.639** | *Snrpn* |

| E20 IUGR | | | | | | | | |
| --- | --- | --- | --- | --- | --- | --- | --- | --- |
| *Cars* | 0.228 | 0.812 | 0.279 | 0.701 | 0.694 | 0.273 | **0.034** | 0.066 |
| 0.309 | *Cdkn1c* | 0.888 | 0.113 | **0.022** | 0.252 | 0.468 | **0.045** | **0.000** |
| -0.065 | 0.038 | *Dnmt1* | **0.000** | **0.039** | 0.284 | 0.478 | 0.144 | 0.204 |
| 0.288 | 0.412 | **0.824** | *Dnmt3a* | **0.001** | 0.105 | 0.812 | 0.957 | 0.871 |
| 0.100 | **0.551** | **0.521** | **0.765** | *Kcnq1* | **0.014** | 0.660 | 0.830 | 0.411 |
| -0.103 | -0.294 | -0.285 | -0.421 | **-0.583** | *Kcnq1ot1* | 0.715 | 0.722 | 0.504 |
| -0.282 | -0.189 | 0.191 | 0.065 | 0.115 | -0.096 | *Peg3* | **0.042** | 0.178 |
| **0.517** | **0.493** | -0.382 | -0.015 | 0.056 | 0.093 | **-0.498** | *Slc22a18* | **0.004** |
| 0.456 | **0.824** | -0.335 | -0.044 | 0.213 | -0.174 | -0.343 | **0.654** | *Snrpn* |

| PN1 Sham + IUGR | | | | | | | | |
| --- | --- | --- | --- | --- | --- | --- | --- | --- |
| *Cars* | 0.785 | **0.000** | **0.000** | **0.000** | **0.028** | **0.000** | **0.000** | 0.103 |
| -0.046 | *Cdkn1c* | 0.460 | 0.136 | 0.731 | 0.439 | 0.836 | 0.807 | **0.043** |
| **0.696** | 0.124 | *Dnmt1* | **0.000** | **0.000** | 0.299 | **0.000** | **0.000** | 0.211 |
| **0.604** | 0.246 | **0.936** | *Dnmt3a* | **0.000** | 0.489 | **0.000** | **0.000** | 0.204 |
| **0.751** | -0.058 | **0.694** | **0.686** | *Kcnq1* | 0.508 | **0.000** | **0.000** | 0.605 |
| **0.357** | 0.129 | 0.173 | 0.116 | -0.111 | *Kcnq1ot1* | 0.305 | 0.103 | 0.550 |
| **0.751** | -0.035 | **0.744** | **0.663** | **0.616** | 0.171 | *Peg3* | **0.000** | 0.330 |
| **0.821** | -0.041 | **0.633** | **0.587** | **0.710** | 0.269 | **0.616** | *Slc22a18* | 0.128 |
| 0.268 | **0.330** | 0.208 | 0.211 | 0.087 | 0.100 | 0.162 | 0.251 | *Snrpn* |

| PN1 Sham | | | | | | | | |
| --- | --- | --- | --- | --- | --- | --- | --- | --- |
| *Cars* | 0.385 | **0.000** | **0.008** | **0.000** | 0.128 | **0.006** | **0.000** | 0.542 |
| -0.218 | *Cdkn1c* | 0.705 | 0.088 | 0.971 | 0.147 | 0.735 | 0.150 | 0.604 |
| **0.759** | 0.096 | *Dnmt1* | **0.000** | **0.000** | 0.699 | **0.000** | **0.020** | 0.489 |
| **0.604** | 0.414 | **0.870** | *Dnmt3a* | **0.001** | 0.798 | **0.001** | 0.075 | 0.428 |
| **0.761** | -0.009 | **0.798** | **0.725** | *Kcnq1* | 0.855 | **0.021** | **0.009** | 0.616 |
| 0.373 | -0.356 | 0.098 | 0.065 | 0.046 | *Kcnq1ot1* | 0.779 | 0.191 | 0.906 |
| **0.624** | 0.086 | **0.740** | **0.703** | **0.540** | 0.071 | *Peg3* | **0.021** | 0.448 |
| **0.835** | -0.354 | **0.544** | 0.430 | **0.595** | 0.323 | **0.540** | *Slc22a18* | 0.280 |
| 0.154 | 0.131 | 0.174 | 0.199 | 0.127 | -0.030 | 0.191 | 0.269 | *Snrpn* |

| PN1 IUGR | | | | | | | | |
| --- | --- | --- | --- | --- | --- | --- | --- | --- |
| *Cars* | 0.292 | **0.008** | **0.034** | **0.001** | **0.002** | **0.000** | **0.001** | 0.232 |
| 0.248 | *Cdkn1c* | 0.363 | 0.409 | 0.384 | 0.450 | 0.940 | 0.283 | **0.010** |
| **0.577** | 0.215 | *Dnmt1* | **0.000** | **0.003** | 0.084 | **0.003** | **0.002** | 0.298 |
| **0.477** | 0.195 | **0.943** | *Dnmt3a* | **0.003** | 0.108 | **0.015** | **0.003** | 0.356 |
| **0.662** | 0.206 | **0.627** | **0.621** | *Kcnq1* | 0.352 | **0.018** | **0.001** | 0.743 |
| **0.638** | 0.179 | 0.395 | 0.370 | 0.220 | *Kcnq1ot1* | **0.011** | **0.027** | 0.668 |
| **0.717** | 0.018 | **0.635** | **0.534** | **0.522** | **0.555** | *Peg3* | **0.007** | 0.439 |
| **0.666** | 0.253 | **0.650** | **0.636** | **0.695** | **0.493** | **0.585** | *Slc22a18* | 0.466 |
| 0.280 | **0.562** | 0.245 | 0.218 | 0.078 | 0.102 | 0.183 | 0.173 | *Snrpn* |
